# Phenotypic switching to hypereosinophilia during cytoreductive therapy for transient abnormal myelopoiesis associated with Down syndrome

**DOI:** 10.1002/jha2.385

**Published:** 2022-01-24

**Authors:** Kenichiro Kobayashi, Asami Watanabe, Shumpei Mizuta, Yoshinobu Nishida, Toshio Heike

**Affiliations:** ^1^ Department of Pediatrics Hyogo Prefectural Amagasaki General Medical Center Hyogo Japan; ^2^ Department of Pediatric Hematology and Oncology Hyogo Prefectural Amagasaki General Medical Center Hyogo Japan; ^3^ Department of Clinical Laboratory Hyogo Prefectural Amagasaki General Medical Center Hyogo Japan; ^4^ Department of Neonatology Hyogo Prefectural Amagasaki General Medical Center Hyogo Japan; ^5^ Department of Pediatric Hematology and Oncology Research Research Institute National Center for Child Health and Development Tokyo Japan; ^6^ Laboratory of Hematology Division of Medical Biophysics Kobe University Graduate School of Health Sciences Hyogo Japan

Transient abnormal myelopoiesis (TAM) is a clonal hematological disorder originating from fetal liver hematopoiesis associated with Down syndrome. The pathognomonic *GATA1* mutation in conjunction with the gene dosage effect of 21 trisomy induces clonal proliferation of megakaryoblasts [1], but little is known for the cell differentiation plasticity, especially phenotypical switching during cytoreductive therapy. A male infant was born through emergency caesarean section due to fetal distress at 30 weeks of gestation with Apgar scores of 1 and 6 at 1 and 5 min, respectively. He weighed 1,338 g and presented with characteristic facial dysmorphism, hepatomegaly and bleeding diathesis. Cytogenic analysis showed trisomy 21. A complete blood count showed a haemoglobin of 7.8 g/dl, a platelet count of 742 × 10^9^/L and a leukocyte count of 326 × 10^9^/L. Morphological findings were as follows: undifferentiated blast, 30%; megakaryoblast, 23%; promyelocyte, 7%; myelocyte, 1%; segmented neutrophil, 1%; monocyte, 2%; lymphocyte, 10% and erythroblast, 26% (top 0 h). Sanger sequencing detected *GATA1* exon 2 nonsense mutation (c.49 C > T) that preferentially induces overproduction of truncated protein isoform, GATA1s. The first round of low‐dose cytosine arabinoside therapy was successful to ameliorate hyperleukocytosis, but we found the emergence of atypical immature myelocytes with large coarse basophilic granules (top 48 h). Despite the continuation of cytoreductive therapies, we found a progression of hypereosinophilia with a following differential count: undifferentiated blast, 5%; megakaryoblast, 5%; neutrophil, 7%; lymphocyte, 12% and eosinophil lineage cells, 71% (top 72 h). He eventually died from multiple organ failure on the 4th day after birth (bottom). We confirmed that *GATA1* mutation ratio was largely unchanged during the clinical course, suggesting that all proliferating cells, including TAM and eosinophilic precursor cells, were clonal in nature. GATA1s can mediate not only eosinophilic cell differentiation but autonomous cell growth via derepressing MYC and E2F transcriptional network [2, 3], we therefore would like to suggest that sustained activation of GATA1s and negative cell selection with chemotherapy had synergistically induced clonal hypereosinophilia. Minakata et al. also reported a female infant who developed late onset clonal hypereosinophilia (on the 18th day of age) during cytoreductive therapy for TAM, which coincided with the exacerbation of liver fibrosis and disseminated intravascular coagulopathy [4]. Given that clonal hypereosinophilia can potently induce end‐organ failure, we would like to stress that vigilant surveillance for immature myelocytes with coarse granules during the cytoreductive therapy is imperative to make an early diagnosis of such a life‐threatening complication associated with TAM.


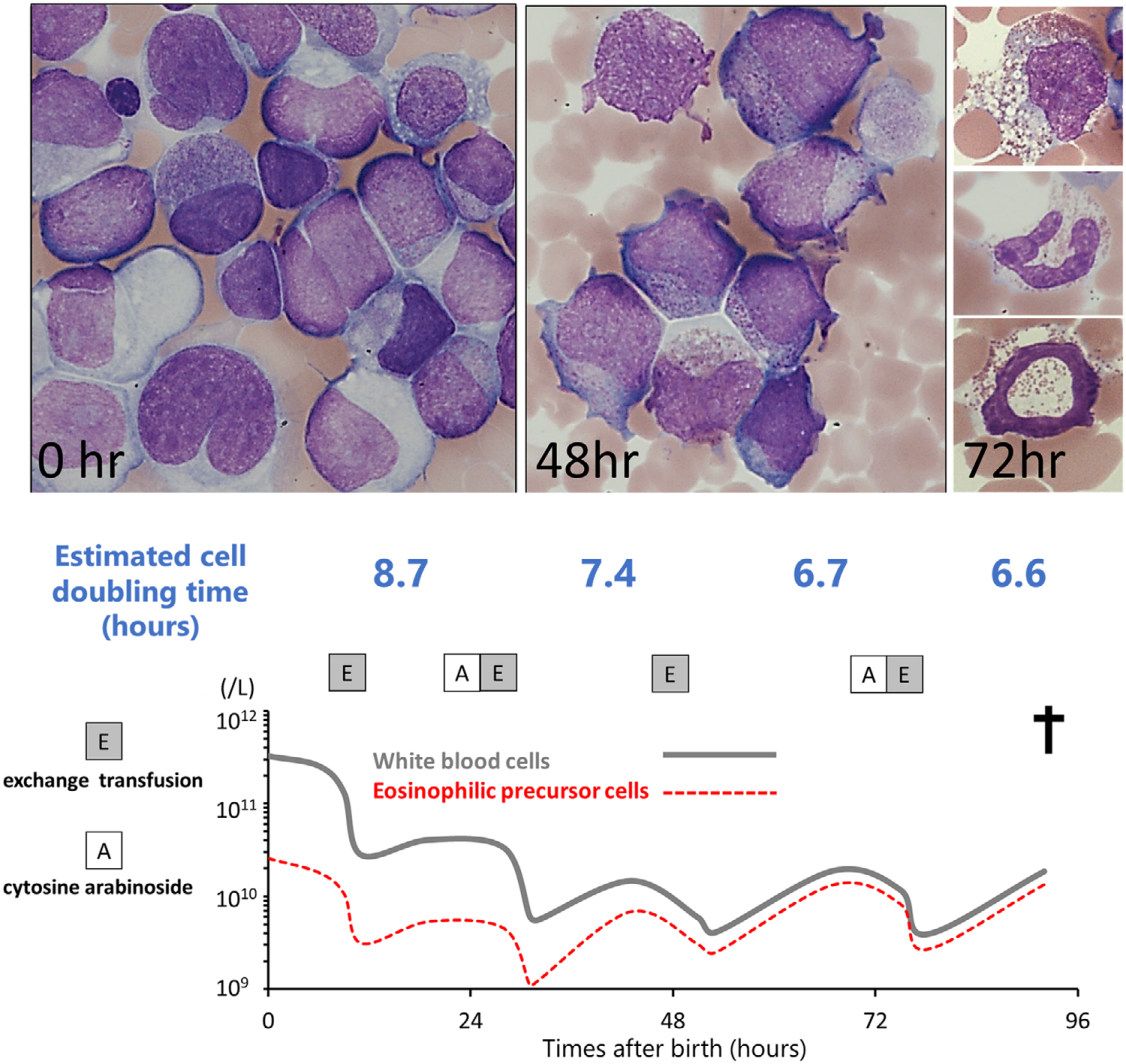


## FUNDING INFORMATION

The authors have no funding to disclose in the preparation of data or the manuscript.

## ETHICS STATEMENT

Written informed consent was obtained from legal guardians for publication of this case report and any accompanying images. The authors affirms that no identifiable images/data of the patients have been utilized in the preparation of the paper.

## CONFLICT OF INTEREST

On behalf of all the authors, the corresponding authors states that there are no conflicts of interest to declare.

Our sincere thanks go to pediatric oncology team especially, Dr. Atsushi Iwai, Dr. Kuniaki Tanaka and Dr. Ikuya Usami.

## AUTHOR CONTRIBUTIONS

Kenichiro Kobayashi and Shumpei Mizuta conceptualised the research scheme, performed data curation and formal analysis, and drafted the paper. Asami Watanabe, Yoshinobu Nishida, and Toshio Heike analysed the data. All authors have approved the final revision of the manuscript for publication.

## Data Availability

The data that support the findings of this study are available from the corresponding author upon reasonable request.
